# Supplementary material for: A multilevel account of hippocampal function in spatial and concept learning: Bridging models of behavior and neural assemblies
Source: Sci Adv. 2023 Jul 21;9(29):eade6903. doi: 10.1126/sciadv.ade6903 (PMC10361583; doi:10.1126/sciadv.ade6903)
Supplement: Supplementary file 1 — Figs. S1 to S5 [file sciadv.ade6903_sm.pdf]

Science Advances
AAAS

Supplementary Materials for

A multilevel account of hippocampal function in spatial and concept learning: Bridging models of behavior and neural assemblies

Robert M. Mok and Bradley C. Love

Corresponding author: Robert M. Mok, rob.mok@mrc-cbu.cam.ac.uk; Bradley C. Love, b.love@ucl.ac.uk

Sci. Adv. **9**, eade6903 (2023)
DOI: 10.1126/sciadv.ade6903

This PDF file includes:

Figs. S1 to S5

Supplementary Materials

Supplementary Figures

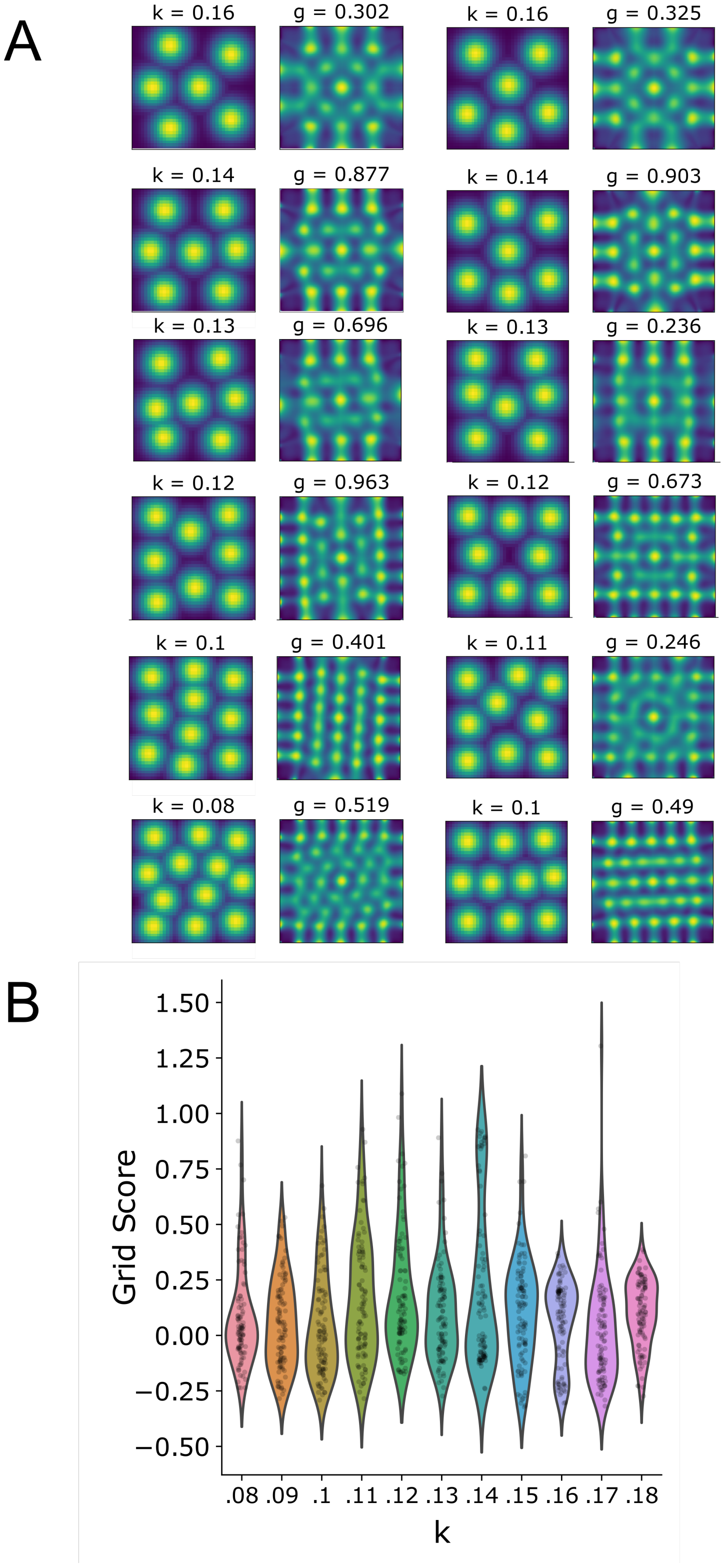

Fig. S1. SUSTAIN-d develops grid-cell like activity patterns with learning. A) Examples of grid cell-like activity patterns and corresponding spatial autocorrelograms after learning across different values of k. B) Distribution of grid scores across different values of k. Recurrent strength is set to 1.0 but similar results were obtained with values of 0.8 and 0.6. k controls the number of flocks or spatial cell assemblies that form.

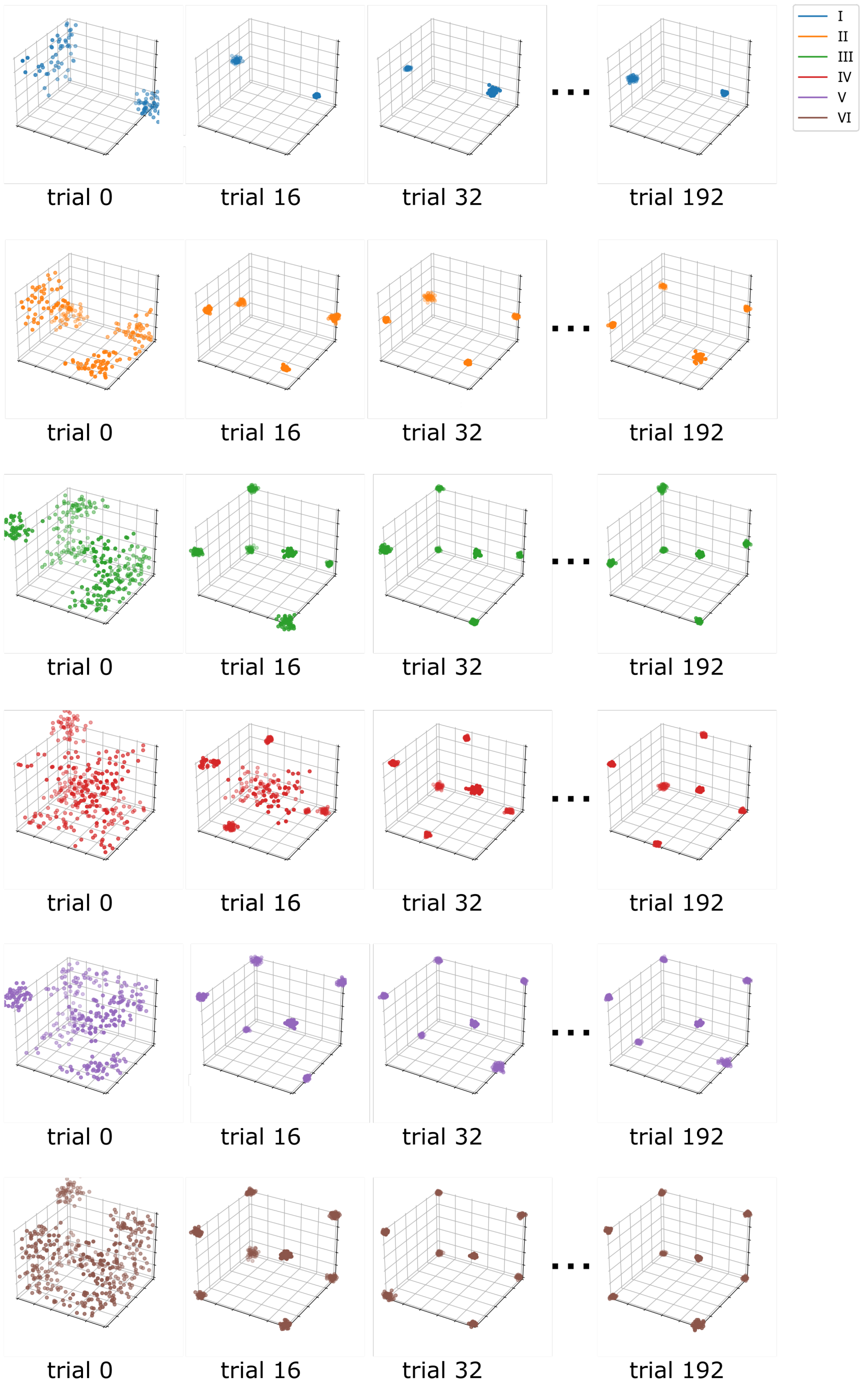
I
II
III
IV
V
VI
trial 0
trial 16
trial 32
trial 192

Fig. S2. Positions of neuron-like units over learning for each concept learning structure from Type I to Type VI (top to bottom) in SUSTAIN-d. To improve visualization, the number of units are sub-sampled from the full population. Neural flocks or virtual clusters form that parallel the number and form of clusters in the higher-level cognitive model SUSTAIN.

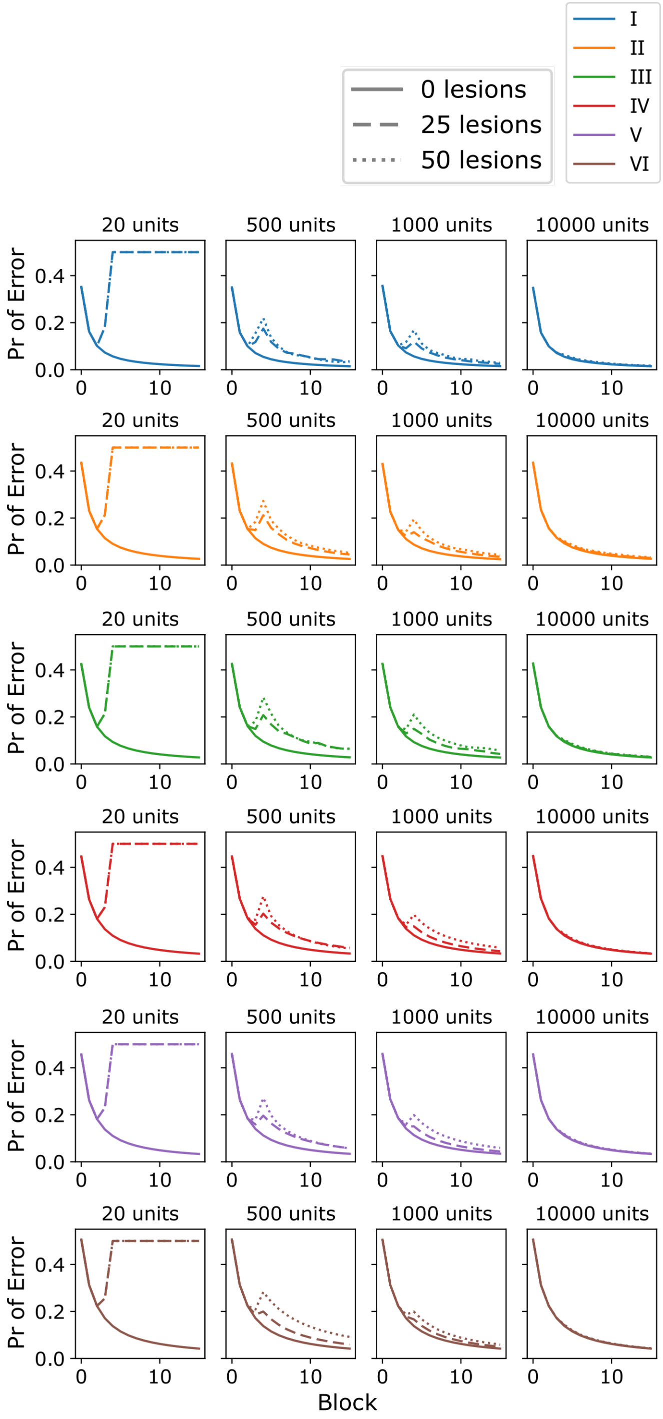

Fig. S3. SUSTAIN-d predicts having many neuron-like units forming neural assemblies (i.e., neural flocks or virtual clusters) makes it robust to individual units failing and noise. With

a large number of units, SUSTAIN-d's aggregate behavior is not noticeably affected by these challenges. Lesion simulations. A 'lesion' event occurs at trial 60 where 0, 25, or 50 units were dropped out of the model. For each concept learning problem, the learning curves are plotted for each model size (i.e., number of units in the model) simulation. The more total units there are providing redundancy and stability, the more robust the model is to lesions. This result holds across all concept learning problems.

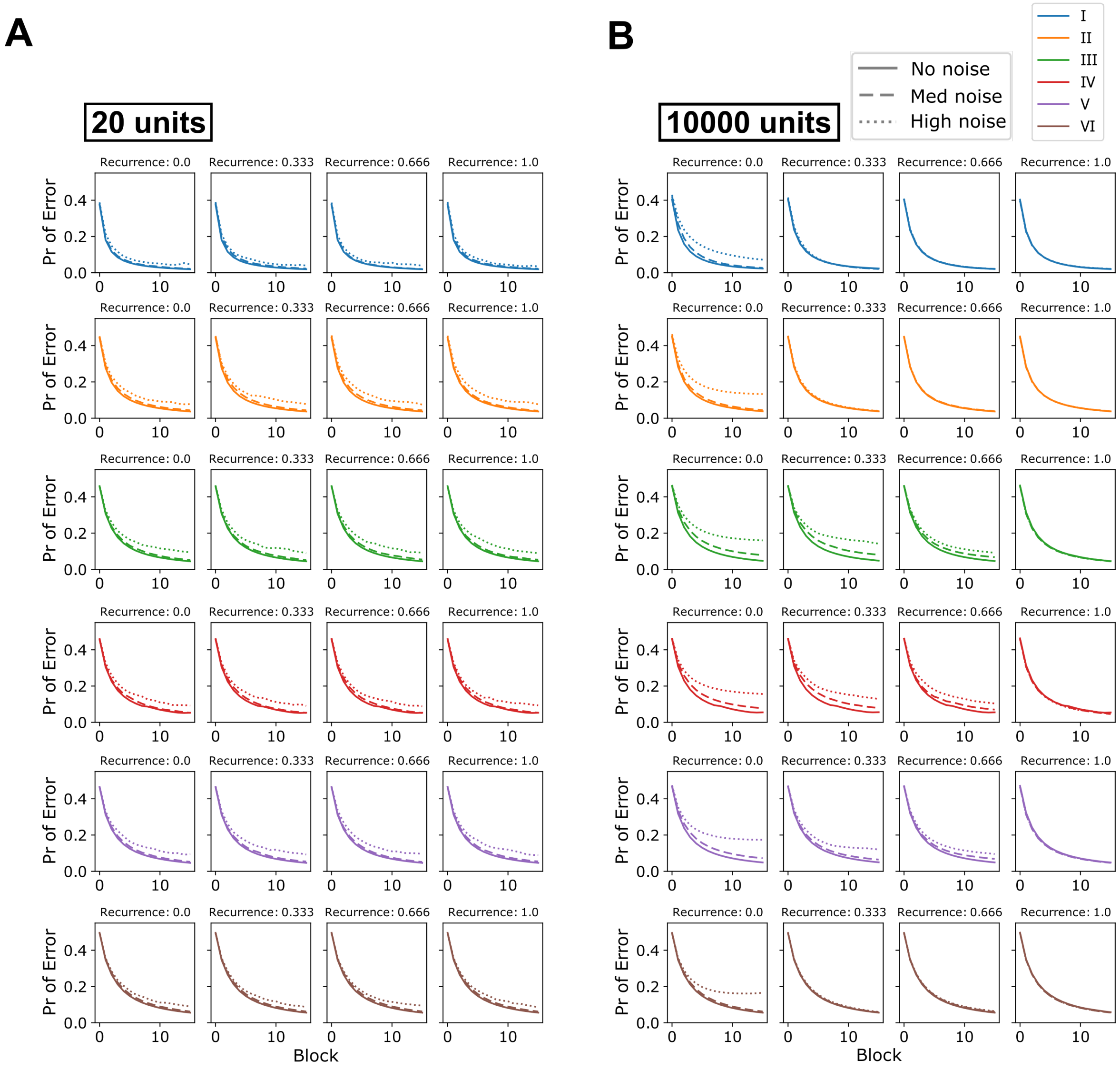
A
B
20 units
10000 units
No noise
Med noise
High noise
I
II
III
IV
V
VI
Recurrence: 0.0
Recurrence: 0.333
Recurrence: 0.666
Recurrence: 1.0
Pr of Error
0.0
0.2
0.4
0
10
Block

Fig. S4. Noise simulations for A) 20-unit and B) 10,000-unit models. Recurrence makes SUSTAIN-d more robust to noise. Learning curves are plotted for each recurrent strength for each problem. Gaussian noise is added to the position of the units on each trial after the first update step (low=0, med=0.5, high=1.0 s.d.). A recurrent step (second update in the learning rule), reduces the effect of noise by pulling individual units' responses towards the mean of flock or the virtual cluster. The greater the recurrent strength, the more the effect of noise is suppressed. Recurrence is especially important when coordinating many units: the strength of recurrence reduces the effect of noise to a greater extent for 10,000-unit models compared to 20-unit models.

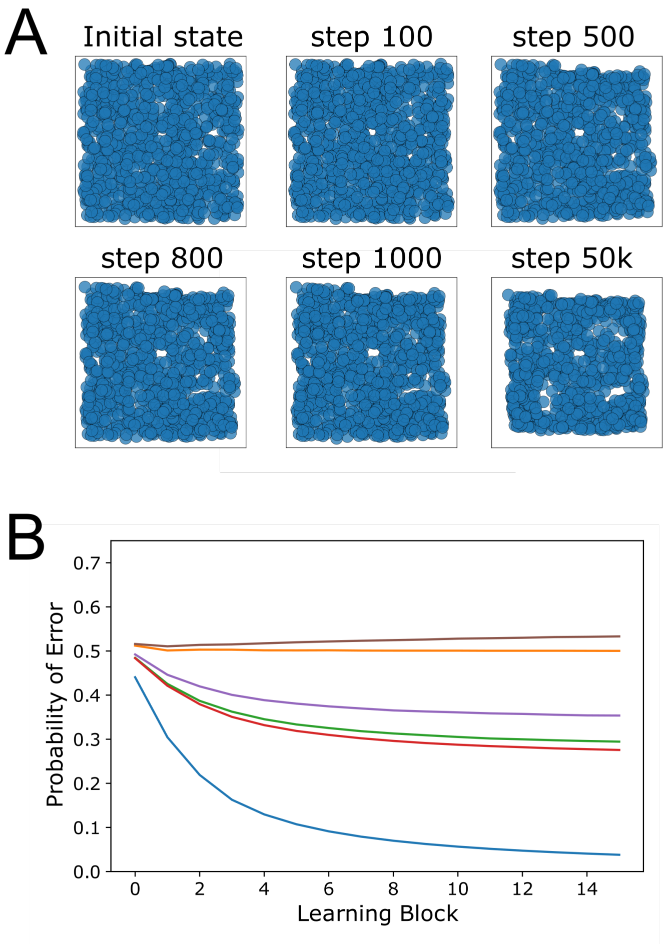

Fig. S5. Control models. A) Spatial simulation with no recurrence leads to no self-organization

of neural assemblies – units' tuning fields simply shift around as the agent moves. B) A prototype model is not capable of capturing Shepard et al.'s (*29*) behavioral learning patterns, and cannot achieve good performance for most problems.
